# Snakin-2 interacts with cytosolic glyceraldehyde-3-phosphate dehydrogenase 1 to inhibit sprout growth in potato tubers

**DOI:** 10.1093/hr/uhab060

**Published:** 2022-01-19

**Authors:** Liqin Li, Chengcheng Lyu, Jing Chen, Yifei Lu, Shiming Yang, Su Ni, Shunlin Zheng, Liping Yu, Xiyao Wang, Qiang Wang, Liming Lu

**Affiliations:** College of Agronomy, Sichuan Agriculture University, No. 211, Huimin Road, Wenjiang District, Chengdu, Sichuan, 611130, China; College of Agronomy, Sichuan Agriculture University, No. 211, Huimin Road, Wenjiang District, Chengdu, Sichuan, 611130, China; College of Agronomy, Sichuan Agriculture University, No. 211, Huimin Road, Wenjiang District, Chengdu, Sichuan, 611130, China; College of Agronomy, Sichuan Agriculture University, No. 211, Huimin Road, Wenjiang District, Chengdu, Sichuan, 611130, China; College of Agronomy, Sichuan Agriculture University, No. 211, Huimin Road, Wenjiang District, Chengdu, Sichuan, 611130, China; College of Agronomy, Sichuan Agriculture University, No. 211, Huimin Road, Wenjiang District, Chengdu, Sichuan, 611130, China; College of Agronomy, Sichuan Agriculture University, No. 211, Huimin Road, Wenjiang District, Chengdu, Sichuan, 611130, China; College of Agronomy, Sichuan Agriculture University, No. 211, Huimin Road, Wenjiang District, Chengdu, Sichuan, 611130, China; College of Agronomy, Sichuan Agriculture University, No. 211, Huimin Road, Wenjiang District, Chengdu, Sichuan, 611130, China; College of Agronomy, Sichuan Agriculture University, No. 211, Huimin Road, Wenjiang District, Chengdu, Sichuan, 611130, China; College of Agronomy, Sichuan Agriculture University, No. 211, Huimin Road, Wenjiang District, Chengdu, Sichuan, 611130, China

## Abstract

The potato tuber is the main nutrient supply and reproductive organ; however, tuber sprouting can reduce its commercial value. Snakin-2 (StSN2) was first reported as an antimicrobial peptide that positively regulates potato disease resistance. Our recent study suggested *StSN2* overexpression inhibited sprout growth, while the sprouting process was accelerated in *StSN2* RNAi lines. Cytoplasmic glyceraldehyde-3-phosphate dehydrogenase 1 (StGAPC1) was identified as a candidate protein that interacts with StSN2 in co-immunoprecipitation/mass spectrometry experiments. Here, we report that the expression levels of *StSN2* and *StGAPC1* decreased during sprouting compared with dormancy. Coexpression of StSN2 and StGAPC1 in bud eyes and apical buds was verified by immunofluorescence analysis of paraffin sections. In addition, interaction of StSN2 and StGAPC1 was confirmed by yeast two-hybrid, co-immunoprecipitation, and split luciferase complementation assays. Overexpression of *StGAPC1* depressed sprout growth, which is similar to the function of *StSN2*, and *StSN2*- and *StGAPC1*-overexpressing lines showed decreased glucose, fructose, and galactose contents. The interaction of StSN2 and StGAPC1 enhanced StGAPC1 activity and decreased its oxidative modification to inhibit sprout growth. Our results suggest that StSN2 plays a regulatory role in tuber sprout growth through interaction with StGAPC1.

## Teaser

**1.** StSN2 and StGAPC1 expression level decreased more in sprouting than dormancy both on transcription and protein process. The coexpression of StSN2 and StGAPC1 in bud eyes and apical buds was verified by immunofluorescence of paraffin sections.

**2.** StSN2 interaction with StGAPC1 was confirmed by *in vivo* and *in vitro* experiments. Overexpression of StGAPC1 depressed sprout growth, similar to StSN2 function.

**3.** StSN2 interaction with StGAPC1 increased StGAPC1 activity and decreased its oxidative modification to inhibit sprout growth.

## Introduction

Potato (*Solanum tuberosum* L.) is the third most important crop species worldwide after rice and wheat. Potato tubers are the main nutrient supply and reproductive organ, and the tuber sprouting process is controlled by environmental, physiological, and hormonal factors. During the sprouting process, potato tubers remobilize storage starch, proteins, and other compounds, and the associated loss of water causes shrinkage [1]. The regulation of sprouting is very important for the timely sowing and long-term storage of the potato tuber. Recent studies have identified certain vital genes that regulate sprouting and have shown that sprouting is strongly delayed in trehalose-6-phosphate (T6P)-accumulating tubers, which is regulated via the SnRK1 signaling pathway [2]. In potato, a decrease in the strigolactone content was in line with the increased rate of tuber sprouting in CAROTENOID CLEAVAGE DIOXYGENASE 8 (ccd8)-RNAi lines [3]. Overexpression of the CENTRORADIALIS gene (*StCEN*) in potato resulted in a lower rate of sprout growth compared with the control by altering the abscisic acid (ABA) and cytokinin contents [4]. Hartmann *et al*. (2011) reported that cytokinin acted with gibberellin (GA) to terminate the tuber dormancy process and activate meristem activity [5].

Members of the plant-specific Snakin/GASA family are involved in growth and many stress responses. Proteins in this family contain 12 cysteine residues, which may be key regions for the physical interaction between GASA proteins and other proteins or active redox reaction sites regulating redox homeostasis in the plant [6]. In *Arabidopsis*, *AtGASA6* plays a role in GA- and ABA-mediated seed germination by promoting cell elongation and increasing hypocotyl length through *Arabidopsis* expansin A1 (EXPA1) function [7]. Snakin-2 (StSN2) was first reported as an antimicrobial peptide isolated from potato. ABA treatment could induce the expression of the *StSN2* gene, while GA treatment could inhibit it [8]. Further research suggested that overexpression of gibberellin stimulated-like 2 (*GSL2,* also known as *StSN2*) in potato conferred resistance to *Pectobacterium atrosepticum* [9]. Our previous study showed that *StSN2* displayed higher expression levels in dormancy than when sprouting [10], and this expression pattern was verified by proteome analysis [11]. Furthermore, *StSN2*-overexpressing tubers exhibited slower sprout growth than RNA interference (RNAi) tubers and altered hydrogen peroxide content, superoxide dismutase, and catalase activities [12]. However, the molecular mechanism by which StSN2 inhibits sprout growth is still unclear.

**Figure 1:**
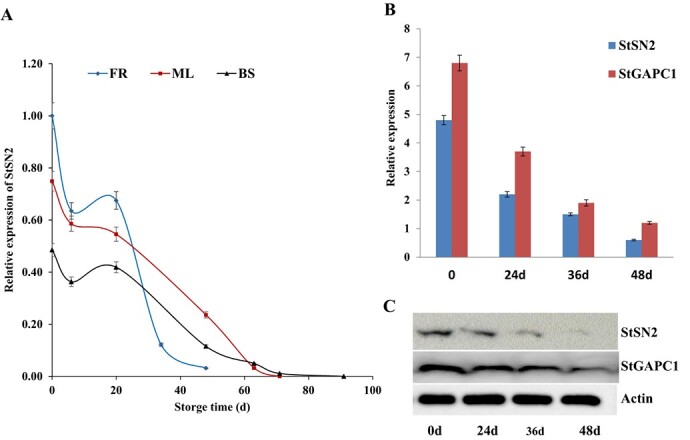
StSN2 and StGAPC1 coexpression patterns at different tuber storage times. **a***StSN2* expression level in three potato varieties during storage. **b** qRT–PCR analysis of *StSN2* and *StGAPC1* gene expression. **c** Western blot assay. 0d indicates harvest, and 24d, 36d, and 48d indicate the time (days) of tuber storage at 20°C. Actin was used as the internal reference.

Glyceraldehyde-3-phosphate dehydrogenase (GAPDH) is one of the key enzymes in glycolysis and is considered to be ubiquitously expressed in various types of cells or tissues. However, recent studies suggested that the mRNA and protein levels of GAPDH varied in response to environmental factors, and the function of GAPDH also represented a diversified division of labor [13]. GAPC is a cytoplasmic GAPDH (EC 1.2.1.12) that specifically catalyzes glyceraldehyde-3-phosphate to 1,3-diphosphoglyceride with NAD^+^(H) as a coenzyme [14]. The downregulation of NAD1-dependent GAPDH (*GAPCp*) triggers an imbalance in the sugar and amino acid ratio [15]. RNAi *of StGAPC1* tubers showed early sprouting and loss of apical dominance and StGAPC1 was also reported to interact with autophagy-related protein 3 to induce cell death in the tuber sprouting process [16].

Here, we identified StGAPC1 as a potential candidate protein that interacted with StSN2 in co-immunoprecipitation (CoIP)/mass spectrometry (MS) experiments (Supplementary Table S1). *StSN2* and *StGAPC1* expression levels decreased in both transcription and protein processes. The interaction between StSN2 and StGAPC1 was confirmed by yeast two-hybrid, CoIP, and split luciferase complementation (SLC) assays. Similar to StSN2, StGAPC1 overexpression delayed sprout growth. In addition, the interaction of StSN2 and StGAPC1 increased the activity of StGAPC1 and decreased its oxidative modification. Our research elucidates the novel roles of StSN2 in delaying sprout growth through interaction with StGAPC1 during the tuber sprouting process.

## Results

### Coexpression of *StSN2* and *StGAPC1* in tuber dormancy and sprouting

**Figure 2:**
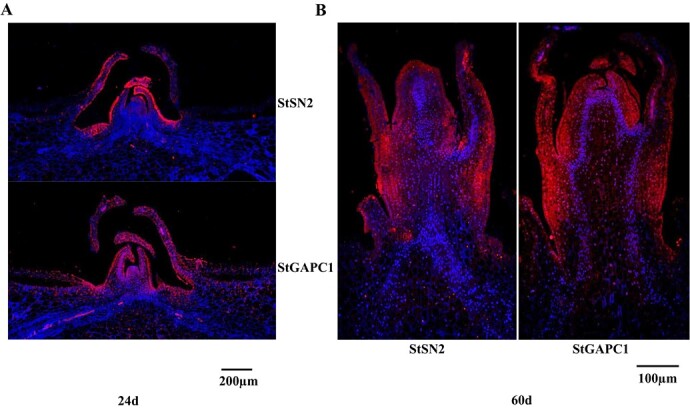
StSN2 and StGAPC1 coexpression patterns in dormancy and sprouting. **a** Longitudinal section of an apical meristem paraffin section during dormancy. **b** Length cutting of an apical bud paraffin section during sprouting. 24d and 60d indicate the time (days) of tuber storage at 20°C. Red indicates a positive signal and blue indicates a negative signal.

To study the relationship between *StSN2* expression level and the sprouting process, three virus-free potato varieties with different dormancy periods were selected, namely, ‘Favourita’ (FR), ‘Mira’ (MR), and ‘Bashu’ 10 (BS), which represent short, medium, and long dormancy, respectively. The real-time fluorescent quantitative PCR (qRT–PCR) results show that *StSN2* was expressed at the highest level in FR and the lowest level in BS after harvest (0 days). With the prolongation of storage in the three varieties at room temperature, the *StSN2* expression level decreased in all three varieties, with the fastest descent in FR and the slowest in BS (Fig. 1a). To elucidate the molecular mechanism by which StSN2 inhibited sprout growth, the target interacting proteins were screened in CoIP/MS experiments. StGAPC1 was one of the possible candidate proteins after the data analysis (Supplementary Table S1), and the qRT–PCR results suggest that the *StSN2* and *StGAPC1* expression levels decreased after 24, 36, and 48 days of storage compared with those at the harvest period (0 days). Moreover, the expression level of *StSN2* decreased 8.0-fold and that of *StGAPC1* decreased 5.6-fold (Fig. 1b). Further western blot assays showed that the abundance of StSN2 and StGAPC1 proteins also obviously decreased after storage, and similar downward trends were observed for these two proteins (Fig. 1c). The immunofluorescence results for the paraffin sections suggest that StSN2 and StGAPC1 were coexpressed in the apical meristem around the bud eye after 24 days of storage (Fig. 2a) and in the apical bud meristem after 60 days of storage (Fig. 2b).

### StSN2 interaction with StGAPC1 *in vivo* and *in vitro*

**Figure 3:**
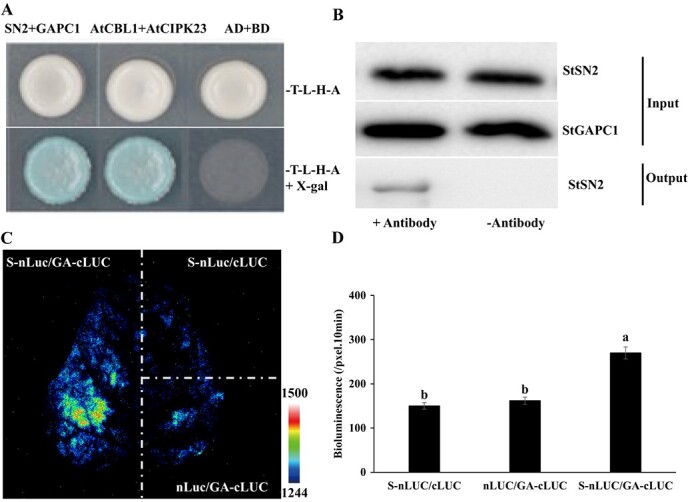
Confirmation of the interaction of StSN2 and StGAPC1. **a** Yeast two-hybrid assay. AtCBL1 and AtCIPK23 were used as positive controls, and AD and BD were used as negative controls. **b** CoIP assay. **c** SLC assay. *S-nLUC/cLUC* indicates *StSN2-nLUC/cLUC*, *nLUC/GA-cLUC* indicates *nLUC/StGAPC1-cLUC*, and *S-nLUC/GA-cLUC* indicates *StSN2-nLUC/St GAPC1-cLUC*. **d** Luciferase activity assay.

Based on the above results, we speculate that StSN2 interacts with StGAPC1 in the sprout growth process. To verify this hypothesis, we performed yeast two-hybrid (Fig. 3a), CoIP (Fig. 3b), and SLC assays (Fig. 3c). The results of these three experiments confirm the interaction of these two proteins. The luciferase activity results also show that the activities of *StSN2-nLUC* and *StGAPC1-cLUC* leaves were higher than those of the two negative control leaves (Fig. 3d). Therefore, we propose that the StSN2–StGAPC1 interaction is necessary in the sprout growth process.

### *StGAPC1* overexpression delays tuber sprouting

**Figure 4:**
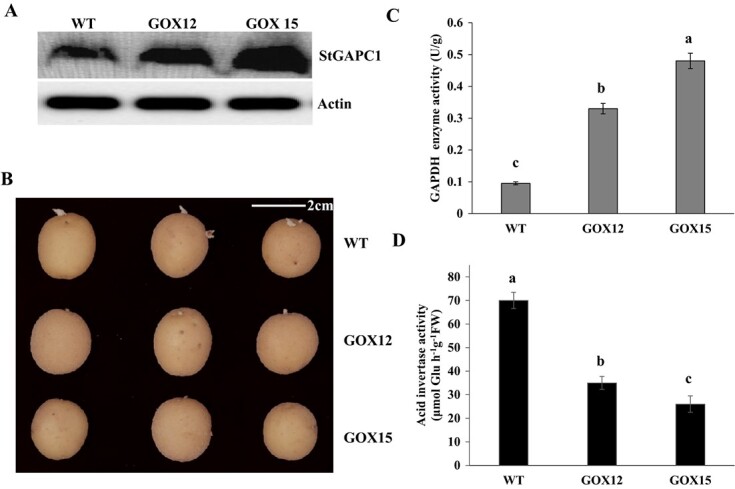
Overexpression of StGAPC1 depresses sprout growth. **a** Western blot assay. GOX12 and GOX15 refer to StGAPC1-overexpressing lines. Actin was used as an internal reference. **b** Phenotype of tuber sprouting. **c** GAPDH activity assay. **d** Acid invertase activity assay. Tuber samples were stored at 20°C for 60 days and used in the experiments.

To evaluate whether StGAPC1 affects sprout growth, we prepared StGAPC1-overexpressing lines (GOX12 and GOX15). The western blot results show that protein abundance was higher in the GOX12 and GOX15 transgenic lines (Fig. 4a). After 60 days of storage, we observed that GOX12- and GOX15-overexpressing tubers exhibited a slower rate of sprout growth than wild-type (WT) tubers (Fig. 4b). Similarly, the GAPDH activity was also higher in these transgenic tubers than in the WT (Fig. 4c). Consistent with *StSN2*-overexpressing lines (Supplementary Fig. S1), acid invertase activity also decreased in the GOX12- and GOX15-overexpressing tubers (Fig. 4d).

### StSN2 and StGAPC1 affect energy metabolism in tubers

**Figure 5:**
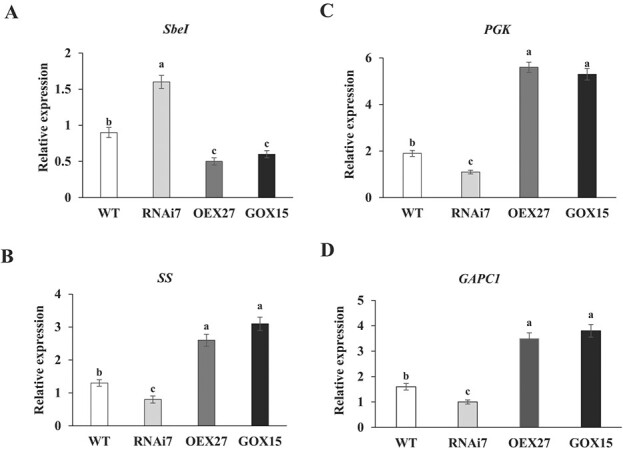
Changes in gene expression in *StSN2* and *StGAPC1* transgenic tubers. **a** Starch branching enzyme I (*SbeI*) expression level assay. **b** Sucrose synthase (*SS*) expression level assay. **c** 3-Phosphoglyceric phosphokinase (*PGK*) expression level assay. **d***GAPC1* expression level assay. Tuber samples were stored at 20°C for 60 days and used in the experiments.

**Figure 6:**
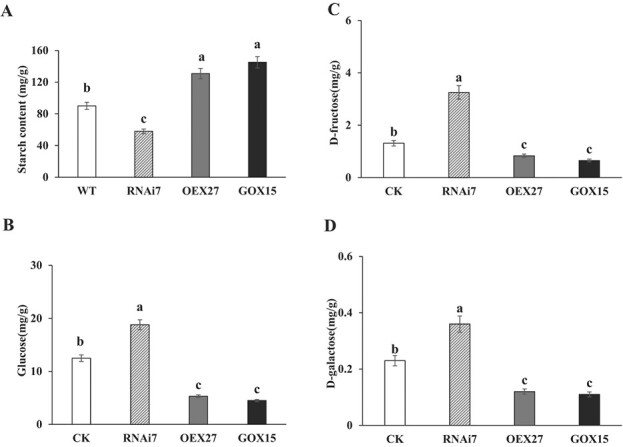
Changes in the carbohydrate metabolism index in *Stsn2* and *Stgapc1* transgenic Tubers. **a** Starch content assay. **b** Glucose content assay. **c** Fructose content assay. **d** Galactose content assay. Tuber samples were stored at 20°C for 60 days and used in the experiments.

To elucidate the effects of StSN2 and StGAPC1 on carbohydrate metabolism in transgenic tubers, qRT–PCR assays were carried out to determine the gene expression of starch branching enzyme I (*SbeI*), sucrose synthase (*SS*), 3-phosphoglyceric phosphokinase (*PGK*), and *GAPC1*. Our results suggest that the *SbeI* expression level decreased by 2- and 1.6-fold in *StSN2*- and *StGAPC1*-overexpressing lines, respectively, but increased by 2-fold in the *StSN2*-RNAi line (Fig. 5a). *SS*, *PGK*, and *GAPC1* showed increased expression in the *StSN2*- and *StGAPC1*-overexpressing lines. The *PGK* expression level increased by 2.9- and 2.7-fold in the *StSN2*- and *StGAPC1*-overexpressing lines, respectively (Fig. 5b–d). We determined four physiological indices, and the results show that the starch content was lower in *StSN2*-RNAi tubers than in *StSN2*- and *StGAPC1*-overexpressing lines during storage at 20°C for 30 days (Fig. 6a). In contrast, the glucose, galactose, and fructose contents were obviously higher in *StSN2*-RNAi tubers (Fig. 6b–d). Thus, it is reasonable to believe that the interaction of StSN2 with StGAPC1 has an important influence on carbohydrate metabolism during tuber sprout growth.

### *StSN2* overexpression increases StGAPC1 activity and decreases StGAPC1 oxidative modification

**Figure 7:**
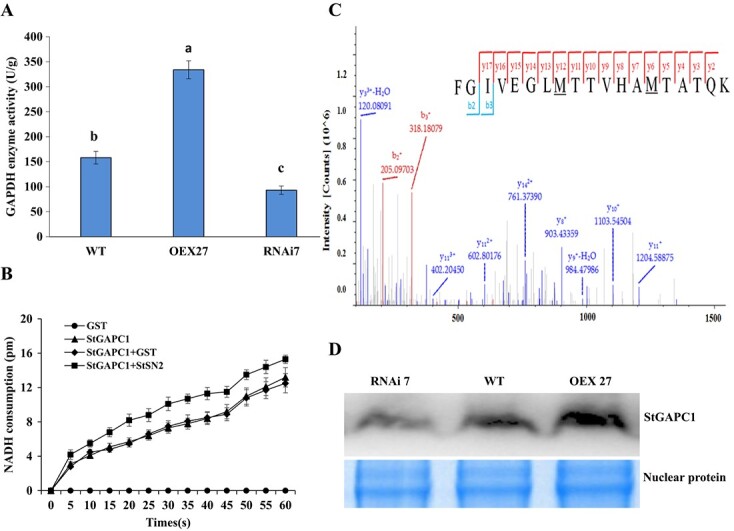
Changes in StGAPC1 activity and oxidative modification in *StSN2*-transgenic tubers. **a** GAPDH activity assay in tuber. **b** StGAPC1 activity assay in *E. coli*. **c** MS analysis of fragments of StGAPC1 protein in WT tubers. Underlining represents the oxidative modification of methionine residues. **d** Nuclear accumulation of StGAPC1. Tuber samples were stored at 20°C for 60 days and used in the experiments.

To clarify the effects of the interaction between StSN2 and StGAPC1 on tuber sprouting, GAPDH activity was tested in *StSN2*-OEX27 and *StSN2*-RNAi 7 transgenic tubers. As expected, the GAPDH activity was 2.11-fold higher in OEX27 tubers than in WT tubers and decreased in *StSN2*-RNAi 7 tubers (Fig. 7a). To determine whether the StGAPC1 activity increased due to interaction with StSN2, GST-tagged StGAPC1 and StSN2 proteins were heterologously expressed in *Escherichia coli* and purified. The results show that the StGAPC1 activity was higher in the sample with mixed StGAPC1 and StSN2 proteins in equal proportions compared with StGAPC1 alone (Fig. 7b). Therefore, the interaction of the two proteins enhances the StGAPC1 activity *in vitro*.

Immunoprecipitation and MS were used to analyze the oxidation sites of StGAPC1 in *StSN2*-OEX27 and WT tubers. The results show that M^179^ and M^185^ were oxidized in WT tubers (Fig. 7c; Supplementary Table S3) but not in *StSN2*-OEX27 tubers; M^235^ was oxidized in both the WT and *StSN2*-OEX27 samples (Supplementary Tables S4 and S5). Thus, the oxidative modification level of StGAPC1 decreased in *StSN2*-OEX27 tubers. Finally, we sought to determine whether the nuclear location of StGAPC1 changed in *StSN2* transgenic tubers because of the low oxidative level. Western blot experiments were performed using tuber nucleoprotein, and the results showed that there was more StGAPC1 protein in OEX27 nuclei than in WT nuclei, and the least amount StGAPC1 protein was in *StSN2*-RNAi 7 nuclei (Fig. 7d). Therefore, we conclude that the interaction between StSN2 and StGAPC1 enhances StGAPC1 activity and decreases its oxidative modification, which possibly changes its nuclear accumulation.

## Discussion

During potato storage, tuber sprouting comprises a series of perplexing physiological and biochemical processes [22]. In recent research, we found that overexpression of *StSN2* inhibited tuber sprout growth, while RNAi lines showed accelerated sprout growth [12]. In the current study, the qRT–PCR results indicate that the expression of *StSN2* is highest in FR (short dormancy variety) and the lowest in BS (long-dormancy variety) after harvest (0 days). The descent speed of *StSN2* was the fastest in FR among the three different dormancy varieties (Fig. 1a). Therefore, we conclude that there is a closely negative correlation between *StSN2* expression and tuber sprouting. This result is similar to that of transcriptome data from tubers in dormancy and sprouting periods [10]. StSN2 expression also decreased at both the transcript and protein levels after storage (Fig. 1b, c). These data indicate that StSN2 acts as a repressor of GA responses, similar to AtGASA5 function. The overexpression of *GASA5* suppressed GA-induced seed germination, and the overexpression lines showed lower germination percentages than the WT in *Arabidopsis* [23]. In contrast, the overexpression of the GA-induced *GASA4* gene promoted flowering and seed germination in *Arabidopsis* [24]. In potato, *StSN1*-silenced lines displayed reduced seedling height and leaf size and changed leaf shape [25]. Similarly, the knockdown of *GSL1* (*StSN1*) and *GSL2* (*StSN2*) was shown to be lethal in potato [9]. In the current study, the *StSN2*-RNAi lines grew similarly to the WT, which might be caused by the 35S promoter used in our transgenic research. The Lhca3.St.1 promoter was used in a previous study [9], and it is known that this promoter is more powerful than the 35S promoter [26], although the 35S promoter is also widely used in potato transgenic research [3, 4, 17].

Although StSN1 and StSN2 have almost identical antimicrobial activity spectra, their sequence similarity is only 38% [8]. These results suggest that StSN1 and StSN2 have diverse functions in the plant development process. Immunofluorescence of paraffin sections indicates that StSN2 is expressed in the active region of cell division, such as in the apical meristem of the bud eye (Fig. 2a) and in the apical bud meristem (Fig. 2b), which implies that it may participate in the process of cell division, similar to StSN1 [27]. To elucidate the molecular mechanism by which StSN2 inhibits sprout growth, a CoIP/MS experiment was performed to identify possible interacting proteins. StGAPC1 was a possible candidate protein among the identified proteins (Supplementary Table S1). In the current study, StGAPC1-overexpressing tubers exhibited a slower rate of sprout growth than WT tubers (Fig. 4b). Previous studies reported that *StGAPC1* knockdown tubers sprouted earlier than WT tubers [16, 28], indicating that StGAPC1 plays a negative regulatory role in sprout growth. Tuber sprouting and the initial growth of buds require energy. Therefore, carbohydrates such as starch, sucrose, and glucose play a vital role in tuber sprouting, and high levels of sucrose and ATP can accelerate the sprouting and growth of buds [22].

Invertase can hydrolyze sucrose to glucose and fructose during tuber sprouting; therefore, potato sprouting is delayed when its gene expression level is suppressed [29]. In our study, acid invertase activity was higher in *StSN2*-RNAi tubers than in WT tubers and lower in both *StSN2*- and *StGAPC1*-overexpressing tubers (Fig. 4d; Supplementary Fig. S1). In addition, sucrose synthase (*SS*), 3-phosphoglyceric phosphokinase (*PGK*), and *GAPC1* all increased in both *StSN2*- and *StGAPC1*-overexpressing tubers (Fig. 5). Consistently, the starch content was higher and the glucose, galactose, and fructose contents were lower in the *StSN2*-RNAi tubers than in the WT tubers (Fig. 6). A similar result was reported in potato, and the fructose, sucrose, and glucose contents also increased in potato leaves overexpressing yeast invertase [30]. These findings suggest that the interaction of StSN2 with StGAPC1 changes energy metabolism during tuber sprouting.

Previous studies have shown that the GASA protein interacts with different proteins to play an important role in plant development. AtGASA4 interacts with the cytoplasmic domain of the receptor-like kinase VH1/brl2 as a polypeptide signal or second messenger to influence leaf venation [31]. In rice, the direct interaction between OsGSR1 and DIM/DWF1 promoted the synthesis of brassinolipids in plants, which indicates that OsGSR1 is indirectly involved in regulating the expression of genes related to the brassinolipid signaling pathway through protein interactions [32]. Similarly, in potato, Nahirñak *et al.* [27] reported that StSN1 could interact with StDWF1, affect the interaction of gibberellin, salicylic acid, and brassinolipids, and participate in hormone balance [27]. In our previous results, no direct interaction was observed between StSN2 and StDWF1 in a yeast two-hybrid experiment, although StSN2 could interact with three peroxidase isoforms involved in peroxidase-regulated lignin synthesis pathways in yeast cells, indicating that StSN2 negatively regulates lignin biosynthesis and hydrogen peroxide accumulation and ultimately inhibits tuber sprouting [12].

In this study, the direct interaction between StSN2 and StGAPC1 was confirmed by yeast two-hybrid, CoIP, and SLC assays (Fig. 3). The *StGAPC1* expression level and GAPDH activity were found to be higher in *StSN2*-overexpressing tubers (Figs 5 and 7a), and the results also show that StGAPC1 activity was higher in samples with the StGAPC1 and StSN2 proteins in equal proportion than in the sample with only the StGAPC1 protein *in vitro* (Fig. 7b). Previous research has shown that plastid glyceraldehyde- 3-phosphate dehydrogenase (GAPCp), along with phosphoglycerate kinase, functions in the production of ATP required for starch metabolism [33]. Piattoni *et al*. [34] also observed that the posttranslational modification process of NAD-GAPDH phosphorylated by SNF1-related protein kinase 1 (SnRK1) plays significant roles in reducing power flux during wheat seed development [34]. Since GAPC contains highly reactive cysteine, it has been regarded as a key redox sensor to regulate energy metabolism [35]. Thus, the interaction of StSN2 with StGAPC1 can promote StGAPC1 activity to change carbohydrate metabolism during tuber sprout growth.

To date, all identified members of the Snakin/GASA family present a signature with twelve cysteine residues in the GASA domain, which is considered to exhibit significant oxidative power [36]. Results have shown that the overexpression of *AtGASA4* and *AtGASA14* suppressed reactive oxygen species (ROS) accumulation, and the GASA domain plays an important role in the hydrogen peroxide response, high salt stress, and GA signal response [37, 38]. *StSN1*-silenced lines exhibited increased levels of ROS, suggesting that StSN1 plays a role in redox equilibrium in potato [27]. According to the above results, we speculate that the interaction between StSN2 and StGAPC1 can affect ROS accumulation to repress sprouting. In plants, GAPC has been reported to suppress ROS as a potential target of hydrogen peroxide [39]. Therefore, overexpressing StGAPC1 may decrease the ROS content to delay sprout growth because the increase in ROS content was an early and relevant event leading to potato sprouting [40]. In *Arabidopsis*, more GAPC protein could be transferred to the nucleus under specific oxidative conditions [41, 42].

Posttranslational modification of GAPC was a required step to enter the nucleus without a nuclear localization signal [43]. In our study, the oxidative modification level of StGAPC1 decreased in *StSN2*-OEX27 tubers compared with WT tubers according to the MS results (Fig. 7c). Correspondingly, the western blot results show that more StGAPC1 accumulated in the nucleus of *StSN2*-overexpressing tubers (Fig. 7d). The low hydrogen peroxide content in *StSN2*-OEX27 tubers [12] might result in a lower oxidative level of StGAPC1 and increased StGAPC1 nuclear accumulation. More experiments will be performed in the future to confirm this conjecture. Interestingly, in *Arabidopsis* seedlings, cadmium treatment increased oxidative conditions in cells and induced cytoplasmic AtGAPC1 protein translocation into the nucleus [44]. In addition, the interaction of GAPCs with phosphatidic acid (PA) afforded signaling to connect carbohydrate and lipid metabolism in *Arabidopsis* [42]. In potato, the relationship of the PA–GAPC1 interaction is unknown; thus, additional research is needed in the future.

**Figure 8:**
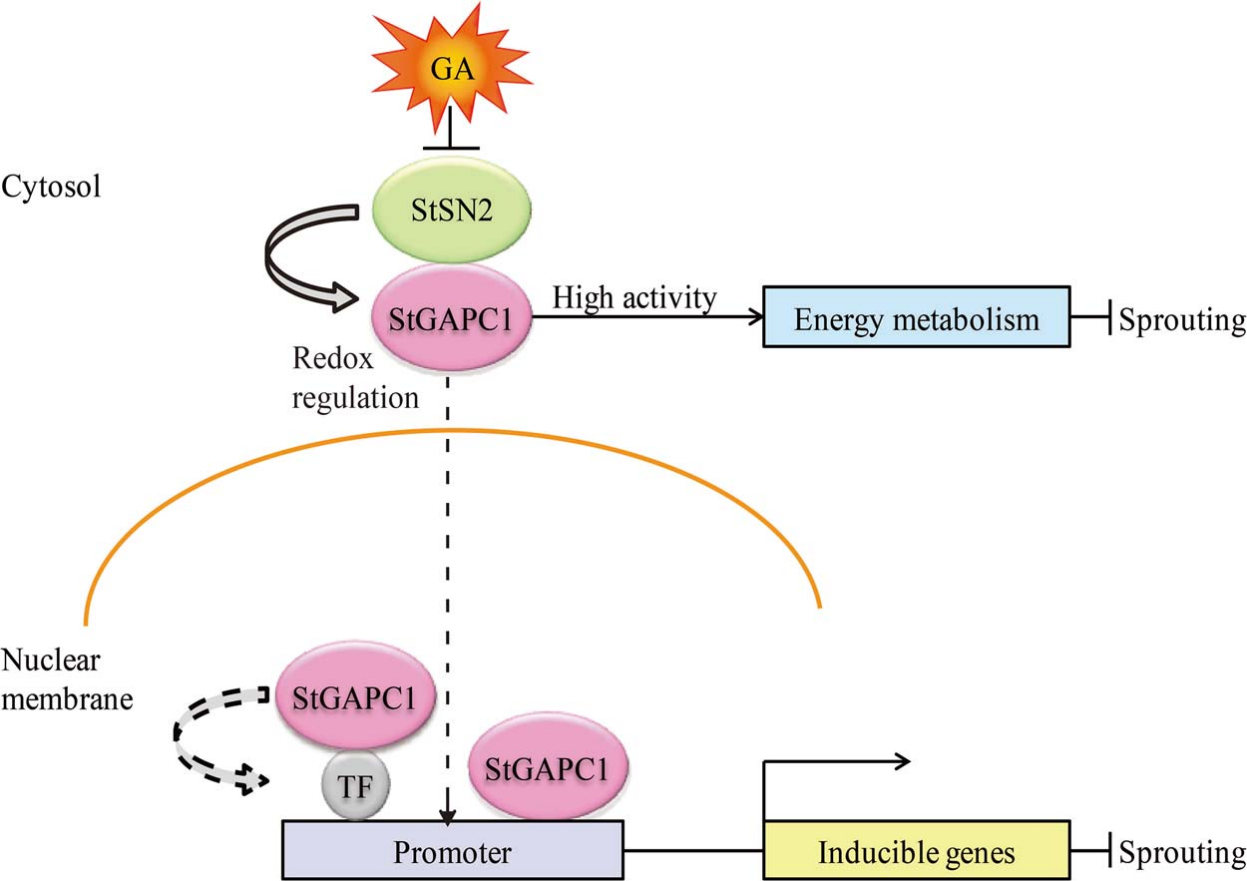
Proposed model of synergistic StSN2 and StGAPC1 inhibition of tuber sprouting. First, GA inhibits *StSN2* expression in tuber sprouting, and the interaction of StSN2 with StGAPC1 increases the StGAPC1 activity to alter the energy metabolism and delay tuber sprouting. Next, the interaction of StSN2 with StGAPC1 decreases its oxidative modification and increases StGAPC1 nuclear accumulation. The nuclear localization of StGAPC1 may combine with the action of some transcription factors or gene promoters to alter vital gene expression involved in tuber sprouting. TF, transcription factor. The dotted line represents speculation.

GAPC1 localized in the nucleus is a transcriptional activator in rice that binds the promoters of some glycolytic genes [ 45]. The latest research also showed that GAPC regulates transcription and physiological responses in heat stress by interacting with a transcription factor (NF-YC10) in *Arabidopsis* [46]. Interestingly, AtGAPC nuclear accumulation might play a role in DNA protection under oxidative stress in *Arabidopsis* [47]. Similarly, lysine ubiquitination and acetylation of AtGAPC also promote its nuclear translocation [ 45, 48]. Amazingly, AtGAPC1 could interact with an E3 ubiquitin ligase (SINAL7); this protein decreased in abundance in *sinal7* mutant and increased in the nucleus of overexpressing lines. It was clear that SINAL7 modulated AtGAPC1 activity and was required for the localization of AtGAPC1 into the nucleus [48]. The above results indicate that GAPC functions differ in different species; however, whether these differences are the manifestation of alienation in the evolution of a species still needs further research. In summary, the nuclear localization of StGAPC1 may act in combination with transcription factors or gene promoters to alter the transcriptional activity of vital genes involved in tuber sprout growth (Fig. 8). Our research contributes to a better understanding of StSN2–StGAPC1 interactions and provides target genes for molecular breeding in potato sprout growth regulation.

## Materials and methods

### Plant material and gene transformation

*Agrobacterium tumefaciens* GV3101 containing the *StSN*2 gene was cultured and shaken in YEB liquid medium with 50 mg/l kanamycin and 50 mg/l rifampicin overnight at 28°C. Microtubers of the ‘Chuanyu 10’ variety that were grown for 12–20 weeks and had a diameter of ~5 mm were cut into slices. Next, the microtuber slices (~1–2 mm) were immersed in the bacterial suspension for 8–10 minutes, cocultured in the dark at 24°C for 36 hours, and transferred onto shoot-regenerating medium for shoot induction. Gene transformation was conducted following a previously described method [17].

### Measurement of physiological indices and enzyme activity

The acid invertase activity was determined by an acid invertase staining method with some modifications [18]. First, the samples were immersed in a fixing solution (2% paraformaldehyde, 2% polyvinylpyrrolidone 40, 10 mM dithiothreitol, pH = 7.0) at 4°C for 1 hour. Then, the samples were placed on an oscillator, vibrated overnight, and washed five times to remove the soluble sugar. A dye solution (25 U/ml glucose oxidase, 0.024% nitrotetrazolium blue, 0.014% phenazine methyl sulfate, 1% sucrose, pH = 6.0) was applied and allowed to react in a 30°C water bath in darkness until blue appeared. The glucose and fructose contents of the tubers were measured using high-performance liquid chromatography as previously described [19]. The NAD-dependent GAPDH activity was assayed as previously described [20].

### Real-time fluorescent quantitative PCR assay

Total RNA was extracted from tubers using TRIzol reagent (Invitrogen, Carlsbad, CA, USA). Complementary DNA was obtained with a reverse transcriptase kit (Thermo, Tokyo, Japan). The qRT–PCR assay was performed following the manufacturer’s protocol using a 7500 Real Time PCR System (Bio-Rad, CA, USA). The relative gene expression levels were calculated using the formula 2^-ΔΔCt^. Elongation factor 1 alpha-like (*EF1αL*) was defined as the internal reference gene, and the primer sequences are provided in Supplementary Table S2.

### Western blot and co-immunoprecipitation assays

For the western blot assay, anti-StSN2 and StGAPC1 antibodies were prepared in rabbits and purified. Ten micrograms of protein was loaded per sample. Next, the nitrocellulose membrane was incubated with purified antibody, and the ECL Select Western Blotting Detection Reagent Kit (Amersham Biosciences, UK) was used to detect target proteins. For the CoIP assay, tuber total protein was extracted, and 10 μl of purified anti-GAPC1 antibody and 20 μl of protein A beads were added; the combination was stored overnight at 4°C and then centrifuged for 5 minutes at 2000 × g to remove the supernatant. Loading buffer was added to the protein A beads before placing in a 100°C water bath for 5 minutes. Then, the beads were centrifuged at 12 000 × g and 4°C for 1 minute, and the supernatant was used for the CoIP assay.

### Immunofluorescence of paraffin sections

Paraffin sections were dewaxed with xylene I and II (5 and 10 minutes,respectively)’ and ‘100, 95, 90, 80, and 70% alcohol solutions (3–5 minutes in each solution)’, and then washed with distilled water for 3 minutes. Antigen repair experiments were conducted using EDTA antigen repair buffer (0.5 M EDTA, pH = 8.0) and were performed in a microwave oven to expose antigenic determinant; the samples were washed three times for 5 minutes with PBS buffer. The slides were covered with 3% BSA for 30 minutes and incubated with antibody overnight at 4°C. The sections were washed according to the method mentioned above and incubated with secondary antibody for 50 minutes in darkness at room temperature. Next, spontaneous fluorescence quenching reagent was added and the slides were incubated for 5 minutes, and the sections were washed in deionized water for 10 minutes before soaking in antifade mounting medium. The detection and collection of images were performed using fluorescence microscopy.

### Yeast two-hybrid and split luciferase complementation assays

For the yeast two-hybrid assay, full-length StSN2 was fused to the vector pGBKT7, and StGAPC1 was fused to pGADT7. Then, both pairs of plasmids *StSN2*- pGBKT7/*StGAPC1*- pGADT7 were cotransformed into yeast cells (AH109). Positive clones were transferred and grown on SD/−Leu-Trp-His plates to clarify the protein–protein interactions, and AtCBL1 and AtCIPK23 were used as positive controls [21]. For the SLC assay, the full-length coding sequences of *StSN2* and *StGAPC1* were fused to the *pCAMBIA1-cLUC* and *pCAMBIA1-nLUC* vectors, respectively, and were cotransformed into *Nicotiana benthamiana* leaves by *Agrobacterium*-mediated transformation. After growing for 3 days, the transformed tobacco was used for the SLC assay. The luciferase signal was detected using a GLOMAX 96 microplate luminometer. The primers used are listed in Supplementary Table S2.

### Mass spectrometry and data processing

Gel pieces obtained from the CoIP assay after SDS–PAGE electrophoresis were rehydrated with 10 ng/μl trypsin and resuspended in 50 mM NH_4_HCO_3_ for 1 hour on ice. Then, after removing the excess solution from the sample, the gel pieces were digested in 50 mM NH_4_HCO_3_ solution with 10 ng/μL trypsin overnight at 37°C. The peptides were collected with 50% acetonitrile/5% formic acid and then dried and resuspended in 2% acetonitrile/0.1% formic acid. The peptides were prepared for tandem MS (MS/MS) using a normalization collision 11 12 energy (NCE) setting of 28, and the fragments were checked in Orbitrap at a resolution of 17 500. A data-dependent procedure alternated between one MS scan and 20 MS/MS scans. The MS/MS data were handled using Proteome Discoverer 1.3 software. The mass error was set to 10 ppm for precursor ions and 0.02 Da for fragment ions. Oxidation on methionine was specified as a variable modification. The peptide confidence was set at high, and the score of peptide ions was set at >20.

### Statistical data analysis

Three biological replicates were performed for all data collection in this study. The data are shown as the means ± standard errors (*n* = 3). The significance of differences between treatments was analyzed using Student’s *t* tests at levels of *P* ≤ .01 and *P* ≤ .05. Different letters represent significant differences at *P* ≤ .05 and/or *P* ≤ .01 among samples in the figures. SPSS 14.0 and Excel statistical software were employed in the data analysis.

## Acknowledgements

We thank Xiaodong Xu from Henan University for providing vectors for the split luciferase complementation experiment.

## Author contributions

L. Li, X.W., and L. Lu conceived and designed the study. C.L., J.C., and Y.L. performed the experiments. S.Y., S.N., S.Z., Q.W. and L.Y. supervised the experiments, analyzed the data, provided critical comments, and/or edited the manuscript; L. Li and L. Lu wrote the manuscript.

## Data availability

All relevant data can be found within the manuscript and its supporting materials.

## Conflict of interest

The authors declare no competing interests.

## Supplementary data

Supplementary data is available at *Horticulture Research* online.

## Supplementary Material

Web_Material_uhab060
