# Supplementary material for: Snakin-2 interacts with cytosolic glyceraldehyde-3-phosphate dehydrogenase 1 to inhibit sprout growth in potato tubers
Source: Hortic Res. 2022 Jan 19;9:uhab060. doi: 10.1093/hr/uhab060 (PMC8972991; doi:10.1093/hr/uhab060)
Supplement: Web_Material_uhab060 [file web_material_uhab060.zip › upplementary Figure 1.docx]

**

**Supplementary fig.1** Acid invertase activity assays among StSN2 transgenic tuber

Tuber sample storage at 20°C for 60d. Different letters indicate significant differences at p < 0.05 among three samples.
